# Experience with oral emergency contraception since the OTC switch in Germany

**DOI:** 10.1007/s00404-016-4253-0

**Published:** 2016-11-28

**Authors:** Marion Kiechle, Miriam Neuenfeldt

**Affiliations:** 1 Lehrstuhl für Gynäkologie und Geburtshilfe, Klinikum Rechts der Isar, Frauenklinik der Technischen Universität München (TUM), Ismaningerstrasse 22, 81675 Munich, Germany; 2 HRA Pharma Deutschland GmbH, Massenbergstrasse 9-13, 44787 Bochum, Germany

**Keywords:** Morning-after pill, Ulipristal acetate (UPA), Levonorgestrel (LNG), Unprotected intercourse, Unintended pregnancy

## Abstract

**Purpose:**

In March 2015, the oral emergency contraceptives levonorgestrel (LNG) and ulipristal acetate (UPA) were released from prescription-only status in Germany. The main research question is to analyse whether the OTC status of oral emergency contraceptives has an influence on the patterns of use.

**Methods:**

All information is based on searches for public domain sources on emergency contraception. Searches were made for scientific publications, statistics, and surveys.

**Results:**

Due to additional active ingredient properties, UPA is superior to LNG in terms of ovulation-inhibiting effect. Since the OTC switch, demand for oral emergency contraceptives has risen by almost 50%, especially at weekends when sexual encounters and thus contraceptive failures are most frequent. However, the age distribution of the users has not changed as a result of the OTC switch. Doctors still play an important role in advising on emergency contraception after the removal of the prescription-only requirement. Pregnancies despite emergency contraception are terminated in more than half of the cases. In federal states with higher rates of use of the morning-after pill, fewer terminations of pregnancy were performed.

**Conclusion:**

As a result of the OTC switch, more women and girls use the morning-after pill after unprotected intercourse and the time between unprotected intercourse and taking the oral emergency contraceptive decreases. This is of great advantage in terms of the mechanism of action. UPA is used more frequently than LNG. Only half of all people aged between 16 and 39 years in Germany are aware of the morning-after pill and 94% of women who had a pregnancy terminated in 2015 did not use any emergency contraception after the unprotected intercourse. In the population, there is still a great need for information and education on contraception and emergency contraception.

## Introduction

As of 15 March 2015, levonorgestrel (LNG) and ulipristal acetate (UPA) are also available in Germany as prescription-free emergency contraceptives. The basis for this decision was the existing evidence on the two active ingredients: if taken soon enough, UPA (ellaOne^®^) and LNG (PiDaNa^®^) effectively delay ovulation, and both have a relatively good drug safety profile [1]. The probability of preventing an unintended pregnancy with oral emergency contraceptives is greatest if they are taken quickly. The low-threshold access in terms of the mechanism of action was thus a further important argument for releasing oral emergency contraceptives from prescription-only status. This decision was a topic of controversy amongst the various partners in the healthcare system.

The purpose of this study is, therefore, to analyse the following questions after 1 year of OTC status:

- Which active ingredients with which mechanisms of action are available?
- What influence has the OTC status of oral emergency contraceptives had on patterns of use?
- Has the user profile changed as a result of the OTC status?
- What role do doctors play since removal of the prescription requirement?
- How high are the pregnancy rates of emergency contraceptive pills and what effects does it have on the pregnancy if the contraceptive action fails?
- Are there effects on the number of pregnancy terminations as a result of the release of oral emergency contraceptives from prescription-only status?
- How well is the population informed about the morning-after pill?

## Methods

The NLM PUBMED literature database was searched for data on oral emergency contraceptives, available active ingredients, and their mechanisms of action. The search terms used were “emergency contraception” and “emergency contraception mode of action”. The first available publication matching the respective search term and all the following publications up to the date of the current search (24.06.2016) were taken into account. A search was made for publicly available online statistics to obtain up-to-date figures on the population, on pregnancies, and on terminations of pregnancy.

To evaluate the market trend, the sales figures for oral emergency contraceptives by active ingredient of IMS HEALTH GmbH & Co. OHG were used [11]. The analysis of the user rate in European comparison is based on the sales figures for European countries of IMS HEALTH GmbH & Co. OHG and on the worldwide population statistics of 15–49-year-old women published in 2012 by the Department of Economic and Social Affairs of the United Nations [13].

The figures on use of oral emergency contraception after unprotected intercourse are based on the figures of IMS HEALTH GmbH & Co. OHG for oral emergency contraceptives sold in 2015 [11], compared with the percentage of women between 14 and 49 years in Germany according to the latest population figures published by the German Federal Office of Statistics in 2014 [14] who are sexually active according to a study on the sexual behaviour of Germans conducted in 2011 by the company Durex [15] and who had experienced a contraceptive failure in the last 12 months according to a European survey by the company BVA Healthcare [22].

The figures on the frequency of use of oral contraceptives in a woman’s life were based on the figures of a representative survey by the German Federal Centre for Health Education on the contraceptive behaviour of adults [16].

An EMNID survey conducted in December 2015 with 1000 interviewees aged between 16 and 39 years was used as the basis for the frequency of sexual intercourse in the course of the week [17]. The percentages of oral emergency contraceptives sold per day of the week at the time of prescription-only status were determined on the basis of a Medimed Prescriber study on the prescribing frequency of the morning-after pill in doctors’ practices on the basis of 14,223 prescriptions in 2010 [18]. The day-of-the-week data after removal of the prescription-only requirement were obtained from the day-of-the-week study on the morning-after pill from October to December 2015 by IMS HEALTH GmbH & Co. OHG [19].

For examination of the effects on the user profiles, the age data at the time of prescription-only status were compared with the data after the switch to OTC status. For this purpose, the figures for the first half of 2010 from the study by medimed GmbH on the age distribution of the morning-after pill users [20] were compared with the figures for July/August 2015 from the market research conducted by HRA Pharma Deutschland GmbH covering 1018 pharmacies [21].

The reasons for using hormonal emergency contraception were obtained from a Europe-wide study conducted by the company BVA Healthcare in 2012 [22]. For this study, 10,983 women were interviewed. The results showed that 2129 of the women interviewed had unprotected intercourse in the last 12 months. The data on use of emergency contraception by these study participants were used.

The figures on unintended pregnancies are based on data from a study on family planning in the lives of women with a focus on unintended pregnancies commissioned by the German Federal Centre for Health Education and published in 2016 [23].

The role of doctors after the switch to OTC status was evaluated on the basis of two sources: on one hand, the analysis of prescriptions for oral emergency contraceptives by IMS HEALTH GmbH & Co. OHG from the year 2015 [24] and on the other hand, a representative online study by the market research institute YouGov with the title “Let’s talk von Frau zu Frau” [Let us talk from woman to woman] in which 1038 women were interviewed in March 2016 [26].

The data on the pregnancy rates on UPA and LNG are based on the meta-analysis published in *The Lancet* in 2010 [27] in which the results of the two available comparative studies on UPA and LNG were taken into account [27, 29].

The data on pregnancies since market authorisation were taken from the pharmacovigilance data on ellaOne^®^ published in 2014 in the journal *Contraception* [32] and from the annual report on reports of adverse drug reactions on ellaOne^®^ from May 2014 to May 2015 (periodic safety update report ellaOne^®^ No. 9) [31].

The number of terminated pregnancies as a percentage of all pregnancies was calculated from the figures on the number of terminations of pregnancy [35] and the number of births in Germany [34], both of which are given on the statistics site Statista.

The pregnancy terminations per 10,000 women by federal state given on Statista for the year 2015 [35] were compared with the rates of use of emergency oral contraceptives by 15–49-year-old women by federal state. To determine the rates of use of oral emergency contraceptives by 15–49-year-old women by federal state, the figures of IMS HEALTH GmbH & Co. OHG on sale of oral emergency contraceptives [11] were compared with the numbers of 15–49-year-old women in the respective federal states [14].

The figures on the use of oral emergency contraceptives by women who had a pregnancy terminated in 2015 were calculated from figures of IMS HEALTH GmbH & Co. OHG on the number of emergency contraceptives sold in 2015 [11], compared with the number of unintended pregnancies in spite of taking oral emergency contraceptives according to the data from the meta-analysis [27] and the number of terminations of pregnancy in the whole of Germany in 2015 given on Statista [35].

The awareness of the morning-after pill in the population was determined by an EMNID survey commissioned by HRA Pharma GmbH in the summer of 2015 for which 1000 men and women were interviewed [37].

## Results

### Available data on active ingredients and mechanisms of action

The available data on the mechanism of action of the two active ingredients are extensive. For the search term “emergency contraception” alone, there are 3318 publications in PUBMED (1947–24.06.2016) and 1354 of which are on the topic of mechanism of action of emergency contraceptives (1953–24.06.2016). From 2002 onwards, there is a sharp increase in publications on the subject mechanism of action. According to the most recent studies, the mechanism of action of oral emergency contraceptives is as follows: if taken early enough, UPA (ellaOne^®^) and LNG (e.g., PiDaNa^®^) delay ovulation [1]. Through agonistic effects on the progesterone receptors of the hypothalamus–pituitary–gonadal axis, UPA and LNG exercise negative feedback and thus suppress the release of FSH and particularly LH by the pituitary [2, 3]. Through the suppression and delay of the LH peak, an important trigger of ovulation is missing, thus resulting in inhibition of ovulation.

UPA, as selective progesterone receptor modulator (SPRM), also has additional effects. Pharmacodynamic studies were able to show that UPA also has ovulation-inhibiting action during the LH increase [4]. When the LH increase occurs in the progesterone receptor, agonist LNG loses its effect. The LH increase (approximately 2 days before ovulation) marks the beginning of the most fertile phase of the cycle. The likelihood of conception on these 2 days is about 30% [5]; however, on account of the variability of ovulation, the time of this phase cannot be predicted [6].

A further additional effect of the SPRM UPA is on the pre-ovulatory progesterone surge. An increased concentration of follicular progesterone together with increased oestrogen and LH levels is a further essential trigger of ovulation [7, 8]. According to pharmacodynamic studies, UPA has an inhibitory effect on the pre-ovulatory progesterone surge [4].

On the follicular level, selectively antagonistic effects of the SPRM UPA are also relevant. Studies in animals showed evidence of the direct antagonistic effects on the follicular level [9]. It was shown on receptor level that activation of the follicular progesterone receptors is necessary for activation of the signal cascade which ultimately leads to ovulation. UPA blocks this signal cascade and can have an additional ovulation-inhibiting action via this direct follicular effect. On the other hand, LNG, as pure progesterone receptor agonist, does not block the follicular, progesterone receptor-mediated signal cascades which lead to triggering of ovulation.

On account of these three additional effects, the SPRM UPA is superior to the progesterone receptor agonist LNG with regard to inhibition of ovulation [4]. UPA is also effective in the most fertile phase of the cycle, during the LH increase, up to shortly before ovulation. The effective window of LNG on the other hand ends 2–3 days before ovulation with the start of the LH increase. The development of UPA as emergency contraceptive thus represents a considerable advance in contraception, particularly on account of its diverse mechanisms of action and low side-effect rate [10].

### Pattern of use

The analysis of the sale figures for oral emergency contraceptives shows, since the switch to OTC status approximately 60,000 packs of hormonal emergency contraceptives per month is dispensed by pharmacies. In comparison with the era of prescription-only status, the market has increased by almost 50% [11]. A look at the development of the market of oral emergency contraceptives from 2014 to 2015 by active ingredient shows a growth in the market from March 2015 onwards [11]. With the removal of the prescription-only requirement in March 2015, there was a sharp increase in the number of morning-after pills sold from about 40,000 per month to approximately 52,000.

Looking at the market growth by active ingredient, we see that the trend set in motion by the gynaecological experts has continued: since 2013 UPA has been the standard for emergency contraception for German gynaecologists [12]. After the switch to OTC status, the superior efficacy of UPA over LNG is still utilised. About two-thirds of the morning-after pills sold and contain the SPRM UPA [11].

Looking at the user rate, we see that in 2015, 3.9% of the women between 15 and 49 years of age in Germany used the morning-after pill [13].

**Fig. 1:**
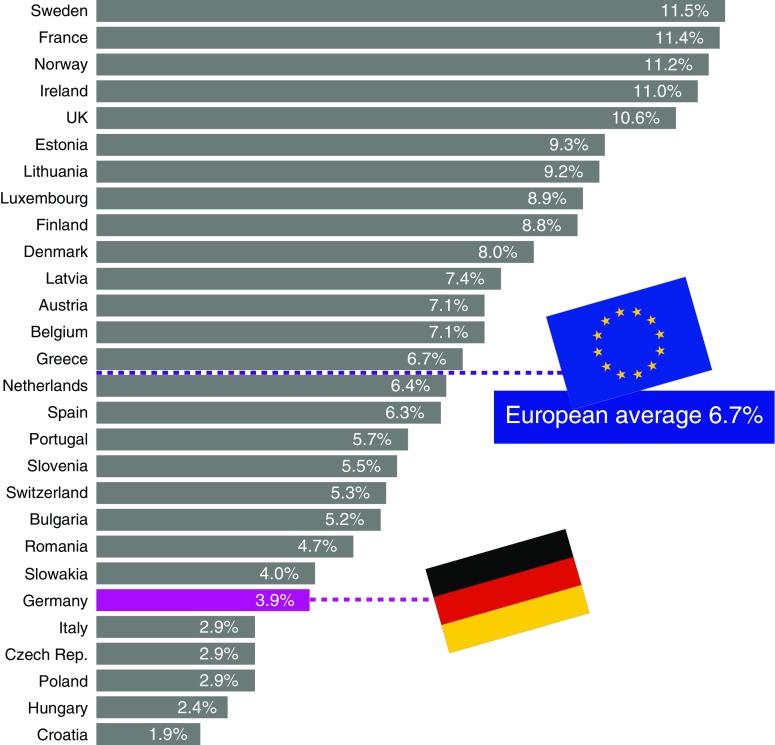
Use of oral emergency contraceptives in Europe as a percentage of the female population of the respective country aged between 15 and 49 years [13]

The European comparison (Fig. 1) shows that in spite of the market growth, oral emergency contraceptives are still used relatively seldom in Germany. The top users in Europe are Sweden, Norway, and France with more than 11% of all 15–49-year-old women. The rate of 3.9% in Germany is well below the EU average of 6.7%.

Estimates show that only 30% of women in Germany use emergency contraceptives after unprotected intercourse to prevent an unintended pregnancy [11, 14, 15, 22] and they usually only use emergency contraceptives once in a lifetime. In a representative survey by the German Federal Centre for Health Education, only 2% of all users reported that they had taken the morning-after pill more than once [16].

**Fig. 2:**
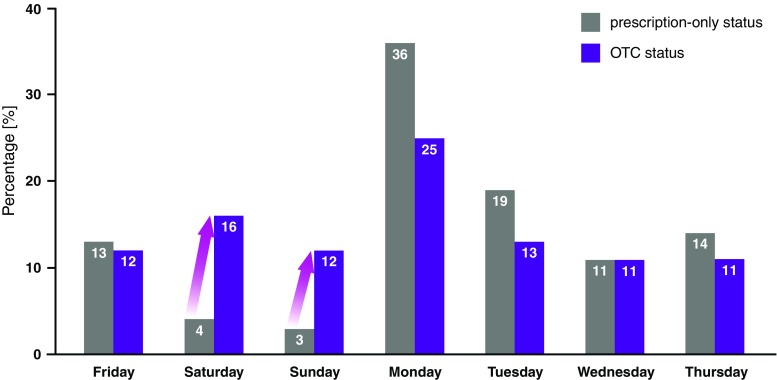
Sales of hormonal emergency contraceptives by day of the week at the time of prescription-only status and after switch to OTC status [18, 19]

According to a recent survey, men and women report that 86% of sexual encounters take place at weekends [17]. The likelihood of contraceptive failures at weekends is correspondingly great. Through the OTC status, women do in fact make use of the rapid access to oral emergency contraception [18, 19]. Thus, the percentage of oral emergency contraceptives sold at the weekend has increased from 7% when the prescription-only requirement was in place to currently 28% (Fig. 2). This means that after unprotected intercourse, more women now make use of the easier access to emergency contraception at weekends.

### User profile

**Fig. 3:**
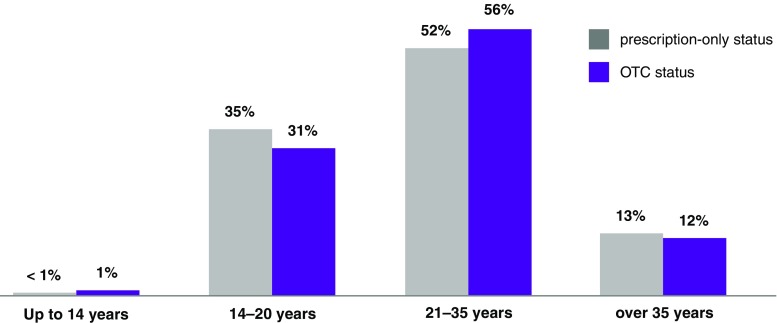
Age distribution of users of oral emergency contraceptives at the time of prescription-only status and after the switch to OTC status [20, 21]

A feared fall in the age of emergency contraceptive users has not occurred: the age of the users of oral emergency contraceptives has not changed as a result of the switch to OTC status [20, 21]. More than two-thirds of users are still over 20 years of age (Fig. 3).

In more than half of the cases, the reasons for taking hormonal emergency contraceptives are failure or forgetting of contraceptive precautions. In a European-wide survey by BVA Healthcare, 39% of the German women interviewed reported condom failure, 34% missed pills, 21% no contraception, 9% a contraceptive pause, and 9% other reasons [22]. Thus, the most common reason for using oral emergency contraceptives is not “no contraception” but failure or forgetting of contraceptives.

Hence, unprotected intercourse occurs mainly as a result of contraceptive failure. This also confirms a survey by the German Federal Centre for Health Education on unintended pregnancies. In this survey, 35.8% of the women who had become pregnant unintentionally stated that they had in fact regularly used contraceptives [23] with 52% using oral contraceptives and 31% using condoms.

### The role of doctors

Doctors—particularly gynaecologists—continue to play an important role for users of oral emergency contraceptives in spite of the fact that the prescription requirement has been lifted. Pharmacies have the option of referring a customer to a doctor at any time, particularly if unclear issues arise during their counselling, e.g., in the case of medical conditions, such as an increased risk of thromboembolic events, severe liver impairment, or epilepsy.

In 2015, 17% of oral emergency contraceptives were dispensed on prescription [24]. Thus, even after the switch to OTC status, some women and girls go directly to a gynaecologist. This is partly because of ignorance about the OTC status of the morning-after pill but also to obtain a prescription for the purpose of reimbursement. In the process of the switch to OTC status for oral emergency contraceptives, the “Fourteenth Ordinance on Amendment of Prescribing of Medicinal Products” [Vierzehnte Verordnung zur Änderung der Arzneimittelverschreibungsverordnung] initiated a corresponding amendment of Article 24a of Book Five of the German Social Security Code (SGB V). This means that doctors can also prescribe non-prescription emergency contraceptives on a statutory health insurance prescription. For women under the age of 20, emergency contraceptives are reimbursable. For women aged 18–20, there is a prescription fee of €5. In spite of regulation of the reimbursability, 17% of oral emergency contraceptives prescribed 12% were prescribed on a private prescription [24].

Further reasons why some women go directly to a gynaecologist after unprotected intercourse may be to have an unplanned pregnancy ruled out with certainty or to use a contraceptive method regularly in the future [25].

The main source of information about the morning-after pill is still the doctor even after the switch to OTC status. This is the result of a recent survey [26]. Almost every second, woman in Germany (44%) who sought information about the morning-after pill consulted a doctor for this purpose. Further popular sources of information are search engines (22%), online health websites (18%), and pharmacy staff (16%).

### Pregnancy rates after emergency contraception

According to the currently available data, the mechanism of action of oral emergency contraceptives is inhibition of ovulation with delay of ovulation [4]. On account of additional active ingredient properties (inter alia stabilisation of the follicle) of the SPRM UPA, the ovulation-inhibiting action of UPA is superior to that of LNG [4]. The high variability of ovulation must be taken into account [6].

Analysis of the two available comparative studies shows that the pregnancy rate on UPA is lower compared with the morning-after pill containing LNG [27]: 0.9% (UPA) vs 2.3% (LNG) when taken within 24 h after the unprotected intercourse, 1.4% (UPA) vs 2.2% (LNG) if taken within 72 h, and 1.3% (UPA) vs 2.2% (LNG) if taken within 120 h. For this reason, the morning-after pill containing UPA (ellaOne^®^) has been the standard for German gynaecologists since 2013 [12].

Analyses (Lancet meta-analysis [27] and Cochrane Review [28]) of the only available two comparative studies [27, 29] show that UPA is superior to LNG.

**Fig. 4:**
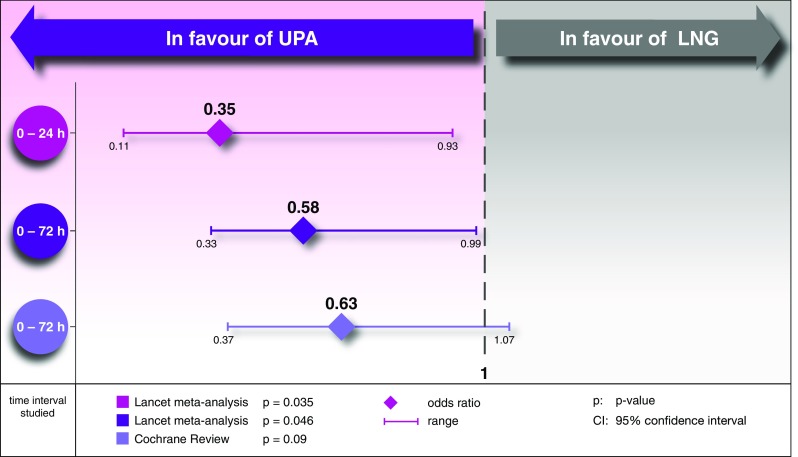
Comparison of effectiveness of UPA and LNG. UPA is superior to LNG, particularly if taken quickly [27, 28]

The superior effectiveness is most marked when UPA is taken within 24 h after the unprotected intercourse (Fig. 4).

In its fact sheet, the WHO for the first time recognizes the superiority of UPA over LNG: the fact sheet states that if LNG is used within 72 h pregnancy can be prevented in 52–94% of the cases. Studies show that, if used within 72 h after unprotected intercourse, UPA can prevent unintended pregnancies in at least 98% of cases [30].

If LNG or UPA is not used soon enough before, ovulation pregnancy is possible in spite of emergency contraception. From the launching of 30 mg UPA for emergency contraception (ellaOne^®^) in October 2009 to May 2015, 604 pregnancies were reported to the manufacturer [31]. The outcome of 207 (34.3%) of the reported pregnancies is not known. At the time of publication of the pregnancy data, 59 (14.9%) of the ellaOne^®^ pregnancies with known outcome were still ongoing and 60 (15.1%) pregnancies resulted in live births. These data show no indications of an increased risk of malformations or complications [31, 32]. Pregnancy in spite of oral emergency contraception is thus not an indication for termination.

### Terminations of pregnancy after emergency contraception

According to the pharmacovigilance databases, 65% of pregnancies occurring after use of UPA were terminated [32].

Just under, 34% of all pregnancies in Germany are unplanned or unintended [23]. More than half of all unintended pregnancies are electively terminated [33]. In Germany, one in eight of all pregnancies is electively terminated [34, 35].

The absolute number of terminations of pregnancy in 2015 did not change significantly compared with 2014: in 2014, 99,715 terminations of pregnancy were performed and in 2015 99,237 [35]. However, when looking at the absolute figures on termination of pregnancy, changes in the population structure must also be taken into account. In 2015, many migrants entered Germany. Nothing can be said about the development of pregnancy terminations in relation to the population until the population figures for the year 2015 become available [36].

**Fig. 5:**
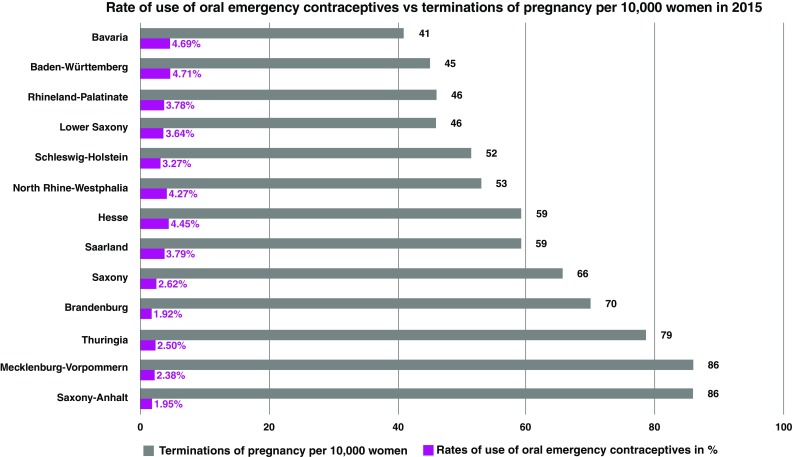
Terminations of pregnancy per 10,000 women in 2015 [35] and the percentage rates of use of oral emergency contraceptives by federal state [11, 14]. Federal states with a higher rate of use of oral emergency contraceptives have lower rates of termination of pregnancy. The rates of use of oral emergency contraceptives in 2015 are calculated on the basis of the data on oral emergency contraceptives from IMS Health and HRA Pharma Deutschland GmbH [11]. The market figures are shown as a percentage of the 15–49-year-old women per federal state on the basis of the population of 2014 [14]

If we compare the figures for pregnancy terminations per 10,000 women in 2015 by federal state [35] with the rates of use of emergency contraceptive pills by 15–49-year-old women [11, 14], the following trend can be identified: in federal states with a low rate of use of emergency contraception, the pregnancy terminations per 10,000 women are higher. In Saxony-Anhalt, 86 of 10,000 women terminated a pregnancy in 2015. The percentage of 15–49-year-old women in Saxony-Anhalt who used the morning-after pill in 2015 was 1.95%. In Bavaria, on the other hand, the pregnancy termination rate is more than two times lower: in 2015, only 41 out of 10,000 women were electively terminated a pregnancy. Compared with Saxony-Anhalt, however, the rate of use of oral emergency contraceptives by 15–49-year-old women was 4.69% and thus more than twice as high (Fig. 5). These figures underline the importance of information and education about contraception and emergency contraception to avoid unintended pregnancies.

### Level of knowledge amongst the population

On the basis of the figures for 2015 on sale of emergency contraceptives [11], the failure rate [27], and terminations of pregnancy [35], it can be estimated that about 94% of the women who electively terminated a pregnancy had not taken any action to prevent a pregnancy after the unprotected intercourse. These figures show that more information and education on contraception and emergency contraception are needed. The lack of knowledge of the option emergency contraception in the population is confirmed by a recent survey of 1000 women and men aged between 16 and 39 [37]: only 53% mentioned the morning-after pill as a possible way of preventing an unintended pregnancy after unprotected intercourse. In the higher income brackets (monthly income €3000–3500), as many as 32% gave termination of pregnancy as an alternative.

## Discussion

The analysis of the available data on emergency contraception shows that as a result of the easier access to oral emergency contraception, more women and girls use the morning-after pill after unprotected intercourse. As a result of the switch to OTC status, the time between unprotected intercourse and the dispensing of emergency contraceptive pills by a pharmacy is shortened. This is of great advantage with regard to the mechanism of action of oral emergency contraceptives. The early use of oral emergency contraceptives increases the likelihood of pre-empting ovulation, and the occurrence of an unintended pregnancy can thus be prevented.

On account of the available evidence showing the superior effectiveness and lower pregnancy risk of UPA compared with LNG, preference is still given to UPA, and the standard medication used by gynaecologists for emergency contraception. The market growth in oral emergency contraceptives is accounted for mainly by UPA.

In spite of the market growth, oral emergency contraceptives are used relatively seldom after unprotected intercourse in Germany. This is shown by comparative data for other European countries, estimates of the frequency of unprotected intercourse, and the figures on unintended pregnancies.

The age profile of the users of emergency contraceptives has not changed as a result of the switch to OTC status. In addition, there is no evidence of an effect on the absolute number of terminations of pregnancy since introduction of OTC status.

It has not been possible to show to date that low-threshold access to emergency contraception alone is able to reduce the number of terminations of pregnancy [38]. It must be taken into account here that there are many reasons for termination of pregnancy. There is no monocausal connection between emergency contraceptives and elective termination of pregnancy. Further important factors which influence the frequency of termination of pregnancy are knowledge and level of education, contraceptive behaviour, accessibility, and costs of contraceptives.

There is, however, happily evidence of a positive trend in Germany: in federal states with a high rate of use of emergency oral contraceptives, the termination rate is lower compared with states in which emergency contraceptives are used less often.

Only half of all young adults in Germany are aware of the possibility of emergency contraception with the help of the morning-after pill. This shows that emergency contraception is still a taboo topic and that intensive educational efforts are still needed. The necessary educational work is additionally impeded by the advertising ban decided on by the German parliament in 2015.

Doctors, who are the most important source of information for the morning-after pill for the general public, also have an information deficit regarding the prescribing of drugs with OTC status. More than 70% of the morning-after pills prescribed were prescribed on a private prescription despite the fact that for under 20 years, the morning-after pill is reimbursable and can be prescribed on a statutory health insurance prescription.

In addition to information and education about emergency contraception, easy and rapid access to oral emergency contraception is of great importance. If a woman wants to prevent an unintended pregnancy using the morning-after pill after unprotected intercourse, early use of oral emergency contraception increases the likelihood of success. If UPA is taken within 24 h after the unprotected intercourse, the pregnancy risk can be reduced to 0.9% [27].

### Core statements

- LNG and UPA are approved in Germany for emergency contraception and have been available without prescription from pharmacies since March 2015.
- UPA prevents unintended pregnancies more effectively than LNG, particularly when used within 24 h after the unprotected intercourse.
- The demand for oral emergency contraceptives has increased by almost 50% as a result of the switch to OCT status.
- Doctors still play an important role in advising on emergency contraception even after the switch to OTC status.
- In federal states with high rates of use of the morning-after pill, there was a trend towards fewer terminations of pregnancy in 2015.
- There is still a great need for information and education on emergency contraception in the population.
